# Electrospun Nanofibrous Membranes for Integrated Dust–Gas Protection in Mining Environments: From Filtration Mechanisms to Multifunctional Design

**DOI:** 10.3390/nano16211411

**Published:** 2026-11-02

**Authors:** Yifan Zhang, Xin Yang, Mingjun Huang, Xin Guo, Yujin Wang, Zhifang Liu

**Affiliations:** 1 Chinese Institute of Coal Science, Beijing 100013, China; 2 School of Mechatronics Engineering, Anhui University of Science and Technology, Huainan 232001, China; 3 Institute of Atomic Manufacturing, International Institute for Interdisciplinary and Frontiers, Beihang University, Beijing 100191, China

**Keywords:** electrospun nanofibrous membranes, integrated dust–gas protection, particulate filtration, toxic gas removal, mining environments

## Abstract

Mining environments present complex respiratory hazards, including both respirable dust and toxic gases. Conventional protective materials typically address only one pollutant type, leaving a gap in integrated protection strategies. This review systematically examines the design of electrospun nanofibrous membranes for simultaneous dust filtration and gas removal. The discussion covers the fundamental mechanisms of particle capture and gas adsorption, the coupling effects between these two functions within a single membrane, and recent engineering strategies ranging from single-polymer systems to composite, hierarchical, and moisture-adaptive architectures. The integration of gas-removal functionalities is addressed through adsorptive materials, metal–organic frameworks for selective capture, photocatalytic degradation, and antimicrobial protection. The review concludes that integrated membrane design must move beyond single-function optimization toward balanced performance in particle capture, gas removal, airflow resistance, and environmental durability. Future progress depends on controlled spatial organization of functional components, humidity-resistant architectures, and standardized testing under realistic multicomponent exposure conditions.

## 1. Introduction

Mining and metallurgical industries remain indispensable for global energy supply and industrial development, yet they continue to expose workers to some of the most hazardous occupational environments [1,2,3]. During drilling, blasting, cutting, crushing, transportation, and smelting operations, large quantities of respirable particulate matter and hazardous gases are simultaneously generated, posing persistent threats to respiratory health and workplace safety. Despite continuous improvements in engineering controls and ventilation systems, occupational exposure remains difficult to eliminate completely, particularly in confined underground environments where pollutant accumulation is common [4].

Respirable dust and toxic gases are the two predominant airborne hazards in mining environments, yet they are often generated concurrently from the same industrial processes. Mechanical excavation and ore fragmentation release abundant respirable coal dust, silica-containing mineral particles, and metal-bearing aerosols, while blasting, diesel-powered equipment, spontaneous combustion, and metallurgical reactions emit hazardous gases such as carbon monoxide (CO), sulfur dioxide (SO_2_), hydrogen sulfide (H_2_S), and nitrogen oxides (NO_x_) [5,6]. In underground workplaces, these pollutants coexist within confined spaces where limited air circulation promotes their accumulation and prolonged worker exposure. More importantly, dust and gaseous pollutants do not simply pose independent risks; their simultaneous exposure can aggravate respiratory injury through synergistic mechanisms, including enhanced particle deposition, toxic species transport, oxidative stress, and inflammatory responses [7]. Consequently, personal respiratory protection in mining environments increasingly requires integrated control of particulate and gaseous contaminants rather than conventional single-function protection.

Current respiratory protective technologies primarily rely on multilayer filter architectures that combine fibrous filtration media for particulate capture with activated carbon or chemically modified adsorbents for gas removal [8]. Particle filtration is generally achieved through interception, inertial impaction, diffusion, gravitational settling, and electrostatic attraction, whereas gas removal depends mainly on physical adsorption, chemisorption, or catalytic conversion. Numerous advanced adsorbent materials, including activated carbon fibers [9], zeolites [10], metal oxides [11], and metal–organic frameworks (MOFs) [12], have been investigated to improve gas adsorption capacity and selectivity. Nevertheless, the stacking of multiple functional layers increases airflow resistance and compromises overall efficiency, while the physical separation of filtration and adsorption components further undermines material utilization [13,14]. High humidity exacerbates these issues by accelerating charge dissipation from electret media and competing with toxic gases for adsorption sites, degrading both particulate and gas removal performance. These limitations highlight the urgent need for multifunctional filter materials capable of simultaneously achieving efficient dust filtration, hazardous gas removal, low pressure drop, and long-term stability under harsh mining conditions.

Electrospinning has emerged as a versatile platform for fabricating nanofibrous membranes with precisely tailored microstructures, including high specific surface area, interconnected porosity, and tunable fiber morphology, as well as multifunctional properties [15]. These structural advantages enable efficient particulate filtration with low airflow resistance, while the ease of incorporating functional materials provides new opportunities for integrating dust filtration and toxic gas removal within a single membrane. For example, a biodegradable dual-functional filtration membrane was fabricated by electrospinning cellulose acetate nanofibers onto a zeolitic imidazolate framework-8 (ZIF-8)-coated cotton fabric, achieving 93.1% particulate filtration efficiency alongside >99% adsorption efficiencies for H_2_S, formaldehyde, and NH_3_ [16]. Recent studies have further demonstrated hierarchical Janus membranes [17], core–shell nanofibers [18], bead-on-string structures [19], and multilayer electrospun composites [20] capable of concurrently achieving high-efficiency particulate filtration and hazardous gas removal with minimal pressure drop. These advances suggest that electrospun nanofibrous membranes represent one of the most promising material platforms for developing next-generation integrated dust–gas protective filters specifically tailored for the complex airborne pollution encountered in mining environments.

**Figure 1:**
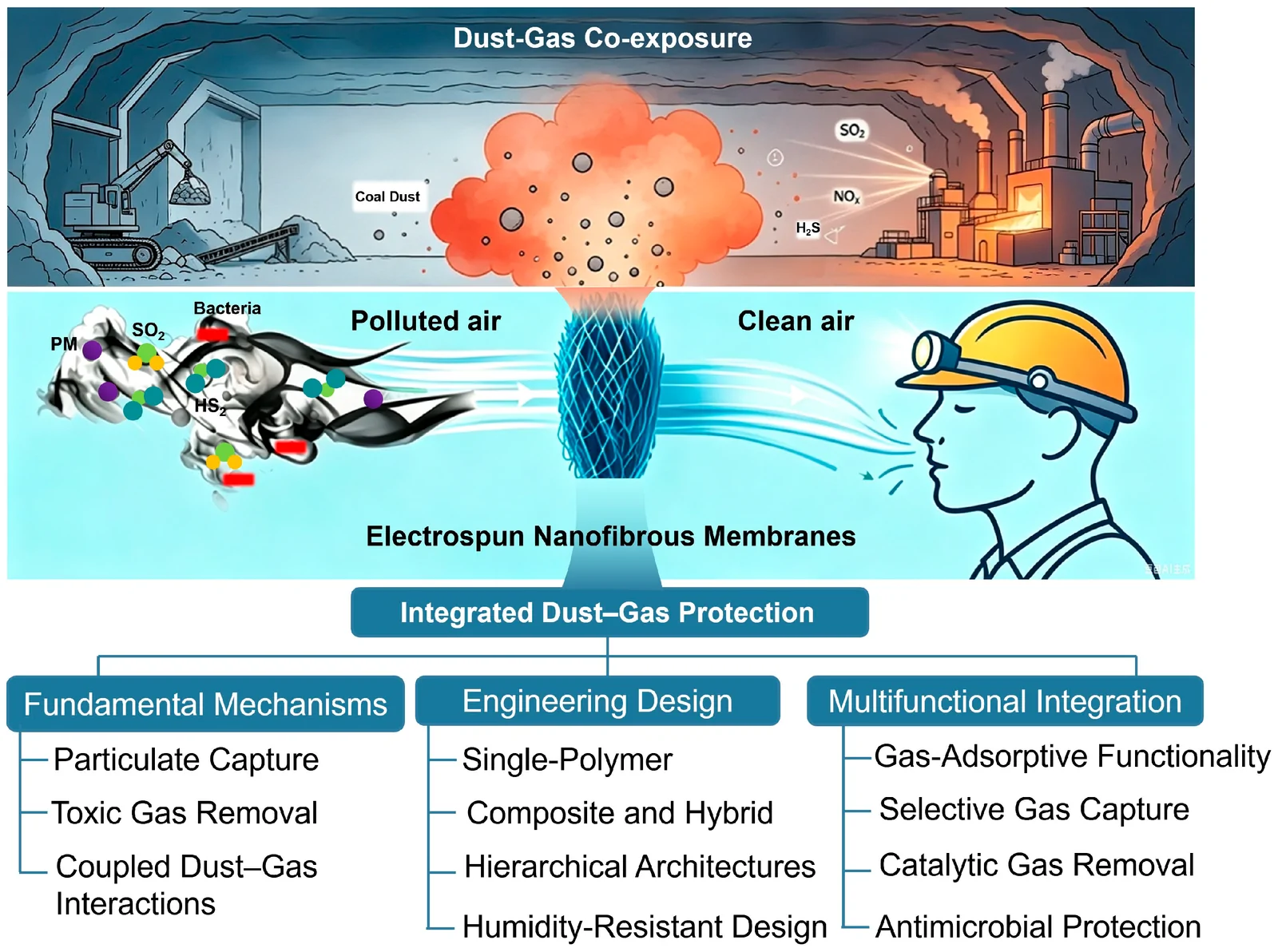
Illustration of integrated dust-gas protection and the outline of this review.

In this review, we provide a comprehensive overview of electrospun nanofibrous membranes for integrated dust-gas protection in mining environments, with a particular focus on bridging fundamental mechanisms, structure design, and multifunctional applications as illustrated in Figure 1. Unlike previous reviews that primarily address particulate filtration, gas adsorption, or electrospun filtration materials independently, this review systematically integrates these aspects within the unique context of mining environments, emphasizing the synergistic design of multifunctional membranes for simultaneous dust filtration and toxic gas protection. We first discuss the fundamental mechanisms governing particulate filtration, gas adsorption, and their coupled interactions to establish the theoretical basis for integrated protective filters. We then review recent advances in electrospun nanofibrous membranes for dust filtration and multifunctional gas protection, highlighting material selection, structural engineering, functional modification, and humidity-resistant design strategies. Finally, key challenges associated with balancing filtration efficiency, gas removal, airflow resistance, and long-term stability under harsh underground conditions are critically discussed, together with future perspectives on intelligent, moisture-adaptive, and self-powered protective systems. By linking filtration mechanisms, membrane architectures, multifunctional integration, and engineering requirements, this review provides a systematic framework for the rational design of next-generation respiratory protective materials for mining and metallurgical applications.

## 2. Fundamentals of Integrated Dust Filtration and Gas Removal

Mining environments often contain a complex mixture of respirable dust and hazardous gases, requiring protective media to simultaneously regulate the transport of particles and gas molecules. Although particulate filtration and gas removal are frequently discussed as independent processes, their governing mechanisms and structural requirements are fundamentally different. Particulate capture is primarily controlled by aerodynamic interactions between particles, airflow, and fibrous structures, whereas gas removal depends mainly on molecular adsorption, absorption, or chemical reactions at active sites. In an integrated protective membrane, these processes are further coupled through pore architecture, mass transfer, surface chemistry, humidity, and contaminant loading. Understanding these fundamentals is therefore essential for the rational design of electrospun nanofibrous membranes that combine high dust filtration efficiency with effective toxic-gas removal and acceptable breathing resistance.

### 2.1. Fundamentals of Particulate Capture in Nanofibrous Membranes

The filtration of airborne particles by fibrous membranes is governed primarily by interception, inertial impaction, Brownian diffusion, sieving, and electrostatic attraction [21,22]. Their relative contributions depend on particle size, fiber diameter, pore structure, face velocity, and the physicochemical properties of both particles and fibers. Large particles are preferentially captured through sieving, interception, and inertial impaction, whereas ultrafine particles are more strongly influenced by Brownian diffusion. Particles within the intermediate size range are generally more difficult to capture, giving rise to the concept of the most penetrating particle size (MPPS) [23]. Electrostatic interactions can further enhance particle capture without proportionally increasing the physical density of the membrane [24].

**Figure 2:**
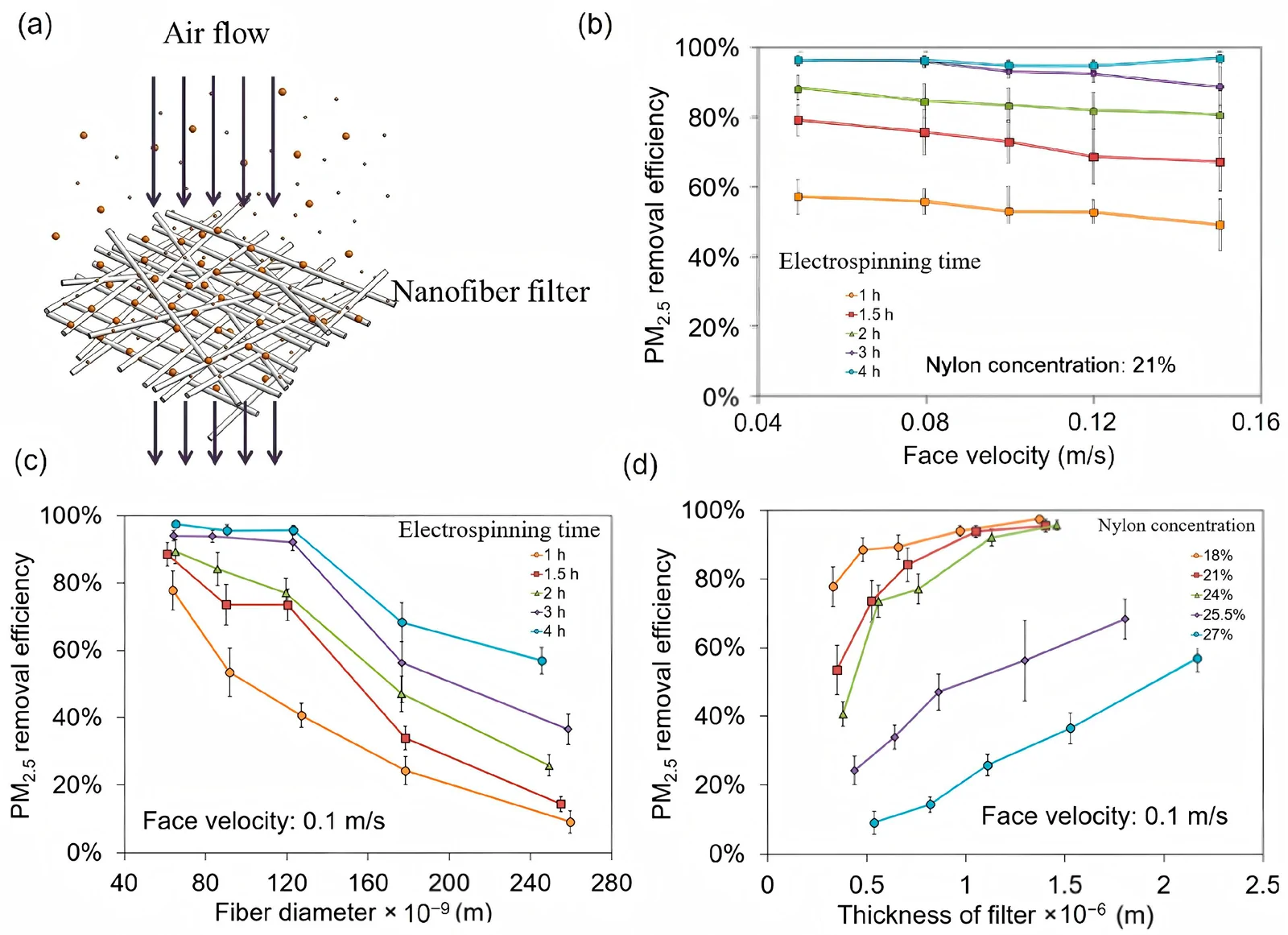
(**a**) A nanofiber air filter for capturing particles with low air resistance, (**b**) PM_2.5_ removal efficiency as a function of face velocity (21% nylon, varying electrospinning time), (**c**) PM_2.5_ removal efficiency as a function of fiber diameter (0.1 m/s, varying electrospinning time), (**d**) PM_2.5_ removal efficiency as a function of filter thickness (0.1 m/s, varying nylon concentration). Adapted from Ref. [26] [Elsevier], Copyright [2020].

Electrospun nanofibrous membranes are particularly attractive because their submicrometer fiber diameters provide a high specific surface area and a large number of interception sites while maintaining an interconnected porous network [25]. Reducing fiber diameter generally enhances particle capture by decreasing the characteristic distance between the airflow streamlines and fiber surfaces. However, maximizing filtration efficiency simply by increasing fiber packing density or membrane thickness inevitably increases pressure drop. This trade-off is particularly important for respiratory protection in mining, where prolonged breathing resistance may reduce wearer comfort and practical usability. As shown in Figure 2, Bian et al. fabricated electrospun nylon nanofibrous membranes and systematically investigated the effects of fiber diameter, thickness, packing density, and face velocity on PM_2.5_ removal efficiency increased with decreasing fiber diameter and increasing membrane thickness, while the relationship with packing density was less straightforward [26]. This study is important because it demonstrates that filtration performance cannot be optimized by a single structural parameter. Instead, the interaction between fiber morphology and airflow transport must be considered. Similarly, electrospun polyacrylonitrile (PAN) nanofiber membranes have been reported to achieve PM_2.5_ removal efficiencies above 90% with a quality factor comparable to or slightly higher than that of a much thicker commercial semi-high-efficiency particulate air (semi-HEPA) filter [27]. These results highlight a key advantage of electrospinning that a thin nanofibrous layer can provide efficient particle capture without requiring the large thickness typical of conventional fibrous filters.

From the perspective of integrated dust–gas protection, however, particulate filtration should not be evaluated solely by initial filtration efficiency. In mining environments, continuous dust loading progressively modifies the pore structure and airflow pathways, increasing pressure drop and potentially altering the accessibility of downstream gas-removal sites. Therefore, an ideal nanofibrous membrane should provide not only high initial particle capture but also controlled dust deposition and sufficient open porosity to preserve gas transport. In our view, the most promising design strategy is not the indiscriminate reduction in fiber diameter, but the construction of hierarchical fibrous architectures that combine a fine-fiber capture layer with a relatively open support layer, thereby balancing filtration efficiency, dust capacity, and airflow resistance.

### 2.2. Fundamentals of Toxic Gas Removal

Unlike particulate filtration, gas removal involves the interaction of individual molecules with the surface or internal structure of an active material. The dominant mechanisms include physisorption, chemisorption, absorption, catalytic conversion, and acid-base interactions, depending on the target gas and functional material [28]. Physisorption is mainly driven by van der Waals interactions and is generally favored by large accessible surface areas and suitable pore structures. Chemisorption and reactive capture rely on stronger interactions between gas molecules and specific functional groups or reactive sites, which can provide improved selectivity but may limit regeneration or long-term stability.

**Figure 3:**
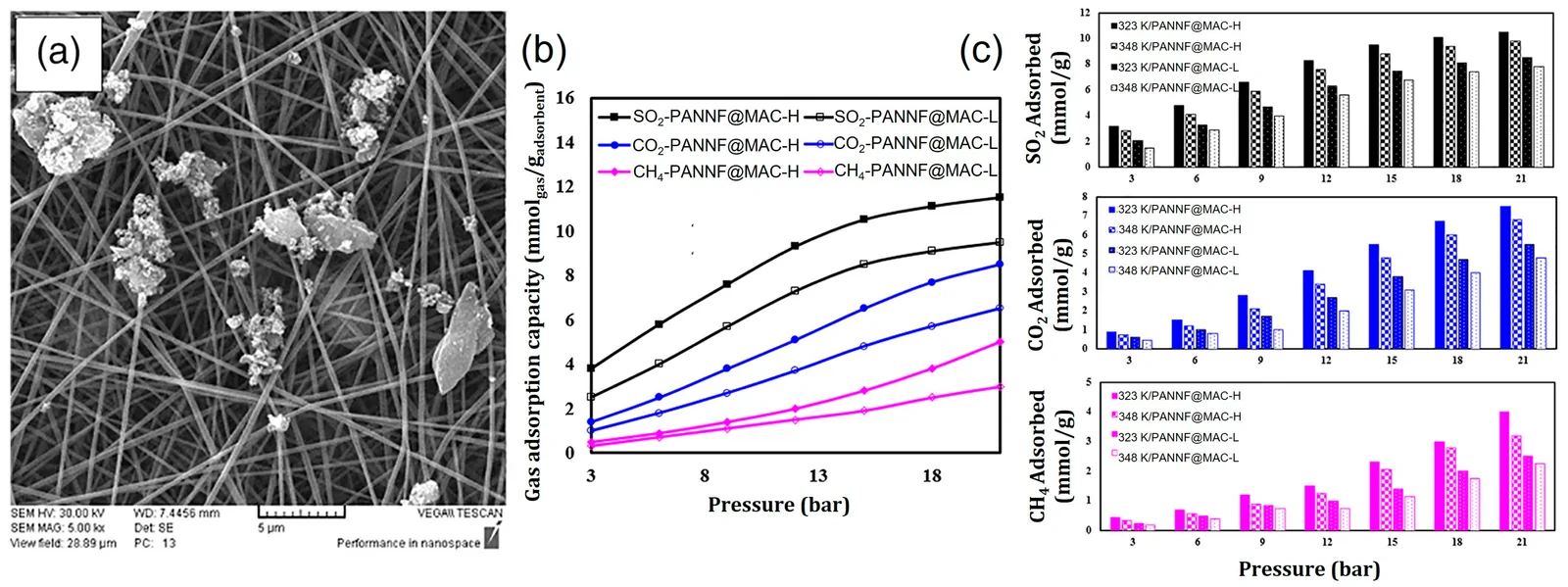
(**a**) SEM images of PANNF/MAC-L, (**b**,**c**) adsorption performance of SO_2_, CO_2_, and CH_4_ on PANNF@MAC-L and PANNF@MAC-H at different temperatures. Adapted from Ref. [29] [Wiley], Copyright [2021].

Electrospun nanofibers are well suited as platforms for gas removal because their high surface-to-volume ratio facilitates rapid mass transfer, while their composition can be readily modified through polymer functionalization or the incorporation of porous adsorbents. As mentioned above, activated carbon, zeolites, MOFs, metal oxides, and reactive nanoparticles can be integrated into or deposited onto nanofibrous structures to provide specific adsorption or reaction sites. This versatility is particularly relevant to mining-related pollutants, which may include acidic gases, sulfur-containing gases, nitrogen-containing gases, and other toxic components. In recent years, electrospun nanofiber systems have consequently been explored for the removal of gases such as NO_x_, CO, H_2_S, SO_2_, CO_2_, and volatile organic compounds (VOCs). As shown in Figure 3a, a representative example is the PAN/activated-carbon hybrid nanofiber system reported by Tajer et al., who fabricated PAN nanofibers by electrospinning and subsequently deposited micro-sized activated carbon particles (MACs) onto the fiber surface via electrospraying [29]. From Figure 3b,c, the incorporation of AC substantially enhanced the adsorption of SO_2_, CO_2_, and CH_4_, with the high-loading PANNF/MAC-H generally exhibiting higher adsorption capacities than the low-loading PANNF/MAC-L. Among the three gases, SO_2_ exhibited the highest adsorption capacity, while the adsorption capacities decreased with increasing temperature, indicating that lower temperatures were more favorable for gas uptake. These results demonstrate that increasing the amount of activated carbon can effectively enhance the gas-removal capability of the nanofibrous membrane, while also highlighting the importance of adsorbent loading and accessible adsorption sites. It also highlights the complementary roles of the two components, with the polymeric nanofiber network providing a lightweight and permeable scaffold and the porous filler supplying abundant sites for gas adsorption. Nevertheless, the effectiveness of this strategy depends strongly on whether the active particles remain accessible to gas molecules after incorporation. Excessive encapsulation of adsorbents inside polymer fibers can reduce available active sites, whereas excessive particle loading may cause agglomeration, mechanical instability, or increased airflow resistance.

More selective gas removal can be achieved using MOFs because their pore chemistry and molecular affinity can be tailored toward specific gases. The broader literature on electrospun gas-purification materials has therefore increasingly emphasized the combination of nanofibrous transport pathways with MOF-based adsorption sites [30,31,32]. The critical design principle is the distinction between adsorbent loading and adsorbent accessibility. A high mass fraction of an active material does not necessarily translate into high gas-removal efficiency if molecular diffusion toward the active sites is restricted. For respiratory protection, the gas-removal layer should therefore maximize the exposure of active sites while maintaining sufficiently short diffusion pathways and low airflow resistance. Surface immobilization, electrospraying, and hierarchical multilayer architectures may consequently be more effective than uniformly embedding large quantities of adsorbents within dense polymer fibers.

### 2.3. Coupled Dust–Gas Interactions and Their Implications for Integrated Protection

In a multifunctional protective membrane, dust filtration and gas removal are not completely independent. Their coupling arises because the same porous structure simultaneously controls particle deposition, gas transport, residence time, pressure drop, and access to active adsorption sites. The addition of gas-active components may modify fiber diameter, surface roughness, pore connectivity, and electrostatic properties, thereby influencing particulate filtration. Conversely, deposited dust can block pores, change local airflow distributions, and cover or deactivate gas-adsorption sites. Consequently, the performance of an integrated membrane cannot be accurately predicted by simply combining the independently measured filtration efficiency and gas-adsorption capacity of its individual components.

**Figure 4:**
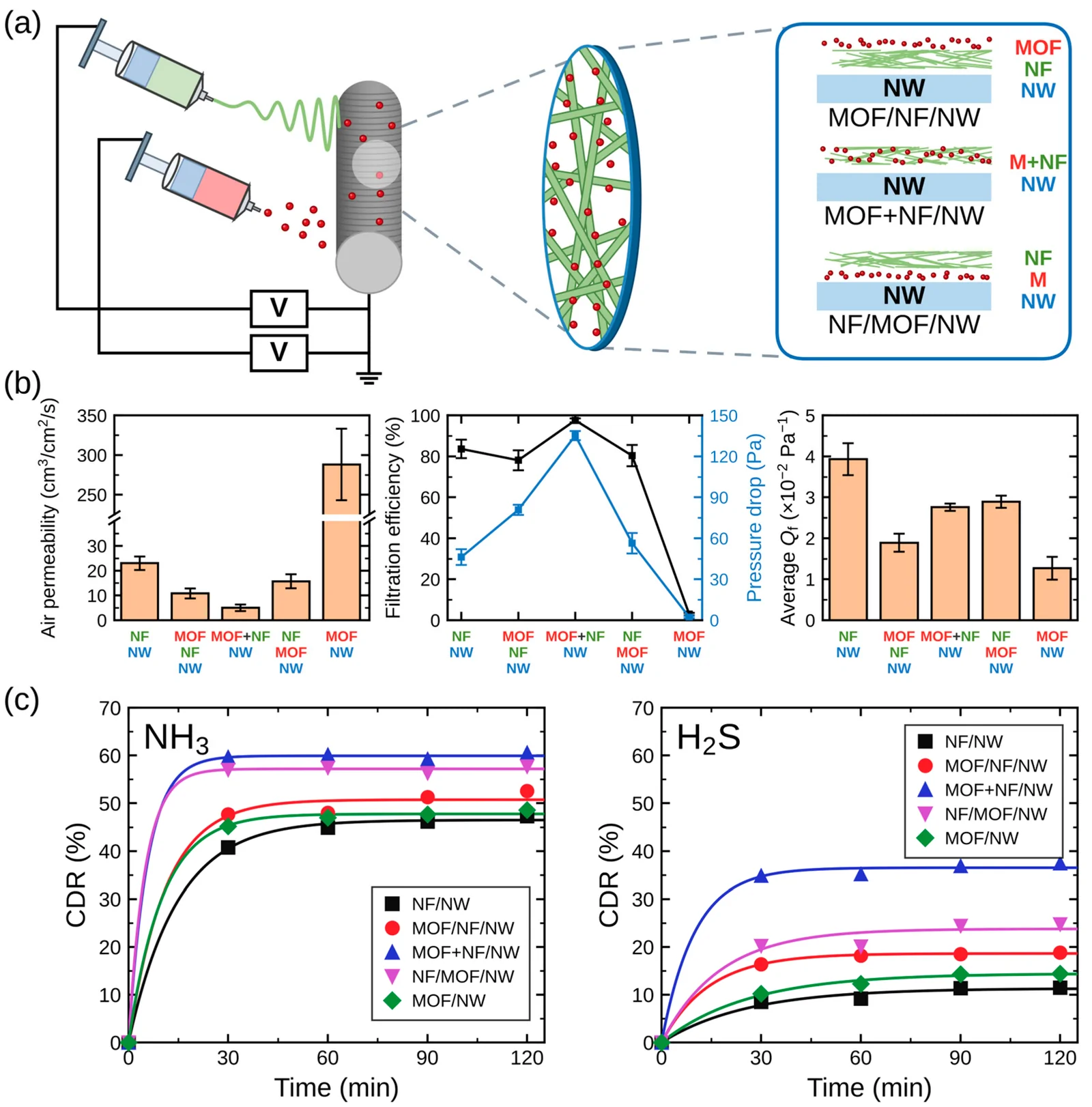
(**a**) Schematic illustration of the fabrication routes and spatial configurations of MOF/NF/NW, MOF + NF/NW, and NF/MOF/NW membranes, (**b**) comparison of air permeability, particulate filtration efficiency, pressure drop, and quality factor for the different membrane configurations, (**c**) toxic-gas removal performance of the different membrane configurations toward NH_3_ and H_2_S. Adapted from Ref. [33] [Wiley], Copyright [2022].

As illustrated in Figure 4a, Kim et al. constructed dual-functional membranes by integrating electrospun poly(vinyl alcohol) nanofibers with Fe-BTC MOF particles in different spatial configurations [33]. These configurations exhibited markedly different air permeability, filtration efficiency, pressure drop, and quality factor, demonstrating that the spatial arrangement of the functional layers directly influences the balance between particle capture and airflow resistance as shown in Figure 4b. From Figure 4c, the MOF+NF/NW configuration achieved a particulate filtration efficiency of 97% while reducing the initial concentrations of NH_3_ and H_2_S by approximately 60% and 35%, respectively. These results demonstrate that the performance of integrated membranes is governed not only by the selection of functional components but also by their spatial organization, which affects pollutant transport, active-site accessibility, and airflow resistance. Thus, effective dust–gas integration requires rational architectural design rather than simply combining filtration and adsorption materials.

This coupling is further complicated by humidity, which is particularly relevant to underground mining environments. Moisture can modify particle adhesion and fiber charging, while competing with toxic gas molecules for adsorption sites, especially on hydrophilic or moisture-sensitive adsorbents [34,35,36]. Accordingly, a membrane optimized under dry laboratory conditions may exhibit altered filtration and gas-removal performance under humid conditions. These effects highlight the need for functional cooperation rather than simple accumulation of filtration and adsorption components. Hierarchical or multilayer architectures, for example, can spatially separate dust capture from gas adsorption, allowing an upstream nanofibrous layer to reduce particulate fouling of downstream active sites while maintaining accessible pathways for gas transport [37]. Recently, Robert et al. designed a bilayered nanofibrous membrane in which an oriented nanofiber layer captures particulates while a downstream random nanofiber layer loaded with silver nanoparticles provides formaldehyde adsorption, demonstrating effective spatial separation of filtration and gas-purification functions [38]. However, excessive functional separation may increase membrane thickness and airflow resistance, whereas hybrid-fiber designs must overcome challenges in active-site accessibility and long-term stability.

These considerations indicate that integrated protective membranes should be evaluated using coupled performance metrics rather than independent single-pollutant measurements. In addition to filtration efficiency and gas-removal capacity, pressure-drop evolution under simultaneous dust-gas exposure, gas breakthrough after dust loading, humidity tolerance, cyclic stability, and performance retention under multicomponent atmospheres should be considered. The key challenge is to develop membrane architectures in which dust capture and gas removal are mutually supportive rather than competitive.

## 3. Engineering Design of Electrospun Nanofibrous Membranes

Building on the filtration mechanisms and dust-gas coupling discussed above, the performance of electrospun membranes can be further tailored through control of material composition, fiber morphology, membrane architecture, and environmental adaptability. Rather than maximizing particle capture alone, effective engineering should simultaneously consider filtration efficiency, airflow resistance, mechanical stability, and resistance to humidity-induced degradation. Recent developments have therefore progressed from conventional single-polymer nanofibers toward composite materials, hierarchical architectures, and moisture-adaptive membranes. These strategies provide the material and structural foundation for subsequently integrating particulate filtration with gas-removal functions.

### 3.1. Single-Polymer Nanofiber Membranes

Single-polymer electrospun membranes based on PAN, PVDF, PVA, PLA, and related polymers remain the most straightforward platform for dust filtration [39]. Their interconnected porous networks and small fiber diameters provide abundant particle-capture sites while maintaining relatively open airflow pathways. However, polymer selection also determines surface polarity, charge retention, mechanical properties, and moisture sensitivity, and therefore directly affects the practical filtration performance. Recent studies have shown that polymer selection alone cannot fully determine the filtration performance of single-polymer nanofibrous membranes, which is also strongly governed by the resulting fiber architecture. For example, Bui-Vinh et al. systematically investigated electrospun polyethersulfone filters with different fiber diameters and found that finer fibers improved filtration performance under comparable pressure-drop conditions, while deposition uniformity and filter configuration further affected the quality factor [40]. More recently, Niu and Chen demonstrated that electrospinning parameters could be deliberately optimized by tuning the applied voltage and spinning time, reducing pressure drop by up to 40.6% while maintaining the target filtration efficiency [41]. These studies collectively indicate that effective design of single-polymer nanofiber membranes requires coordinated control of polymer properties, fiber dimensions, deposition configuration, and electrospinning parameters rather than polymer selection alone.

**Figure 5:**
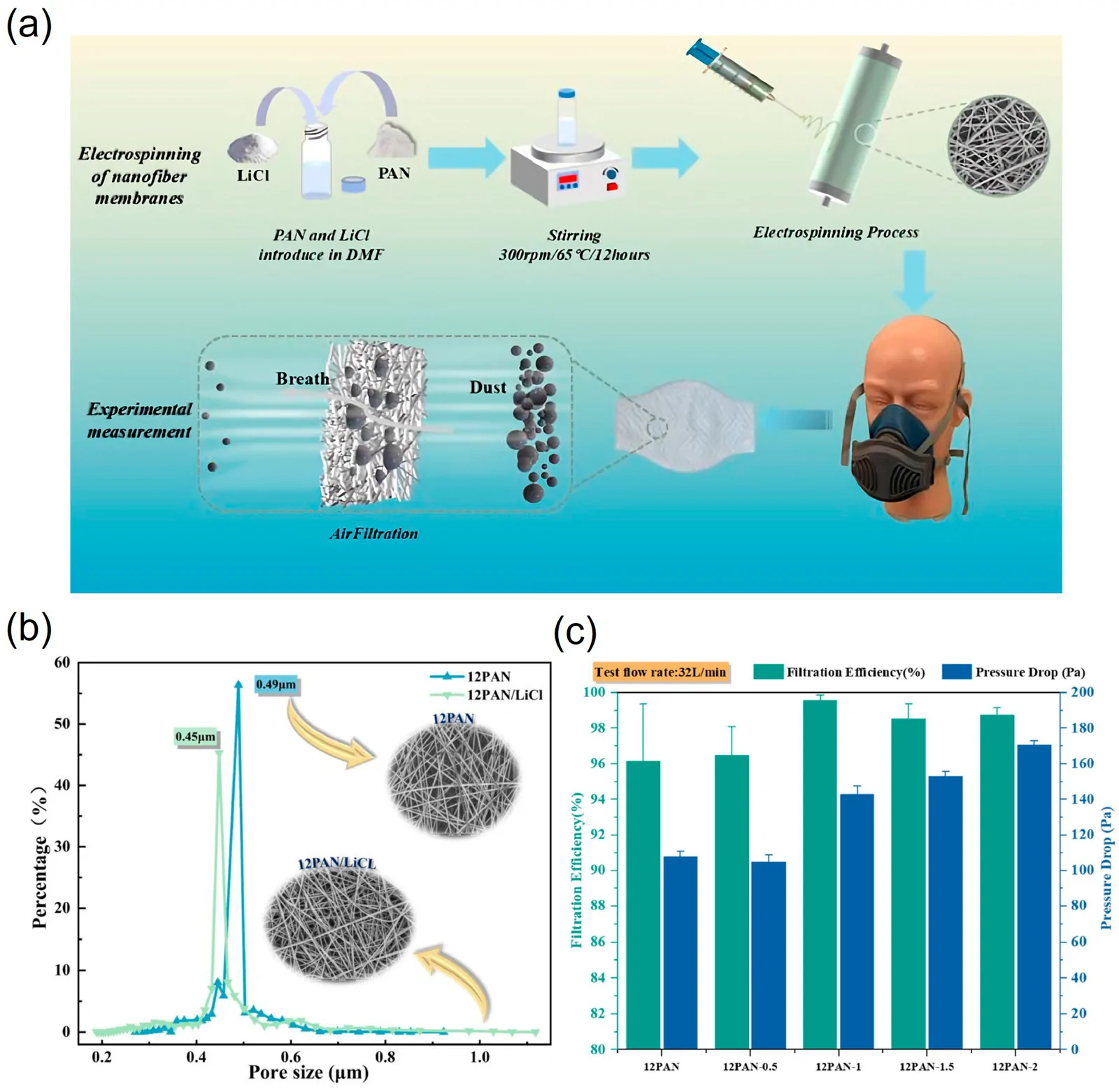
(**a**) The process of preparing the nanofiber membrane, (**b**) pore size distributions of the PAN nanofiber membrane and the PAN/LiCl nanofiber membrane prepared from a 12 wt% PAN spinning solution, labeled as 12PAN and 12PAN/LiCl in the figure, respectively, (**c**) filtration performance of nanofiber membranes at flow rate of 32 L/min. Adapted from Ref. [42] [Elsevier], Copyright [2026].

As shown in Figure 5, a particularly relevant example for mining applications is a PAN/LiCl nanofibrous membrane that achieved a 99.54% filtration efficiency for 0.3 μm particles with a pressure drop of only 142.8 Pa [42]. The incorporation of LiCl modified the electrospinning process and fiber morphology, leading to a more favorable balance between particle capture and airflow resistance. Such results indicate that even within a nominally single-polymer system, relatively simple regulation of the spinning solution can substantially alter the nanoscale structure and filtration behavior. Nevertheless, single-polymer membranes generally provide limited freedom to independently tune filtration performance, mechanical strength, and environmental durability. It is noted that environmental durability refers to the ability to retain structure and function under the humidity, elevated temperature, and chemical or oxidative exposure that characterize mining atmospheres, rather than to any single material property. This limitation motivates the development of composite and hybrid systems.

### 3.2. Composite and Hybrid Nanofiber Membranes

Composite strategies introduce additional degrees of freedom by combining different polymers or incorporating functional nanoparticles into electrospun fibers. Polymer blending can simultaneously modify fiber morphology, mechanical properties, surface chemistry, and charge characteristics, whereas nanoparticles such as TiO_2_, SiO_2_, and graphene derivatives can enhance particle-fiber interactions or alter the porous architecture [43,44,45]. Importantly, the benefit of an additive depends not simply on its intrinsic properties but on its dispersion, loading level, and accessibility within the nanofibrous network.

**Figure 6:**
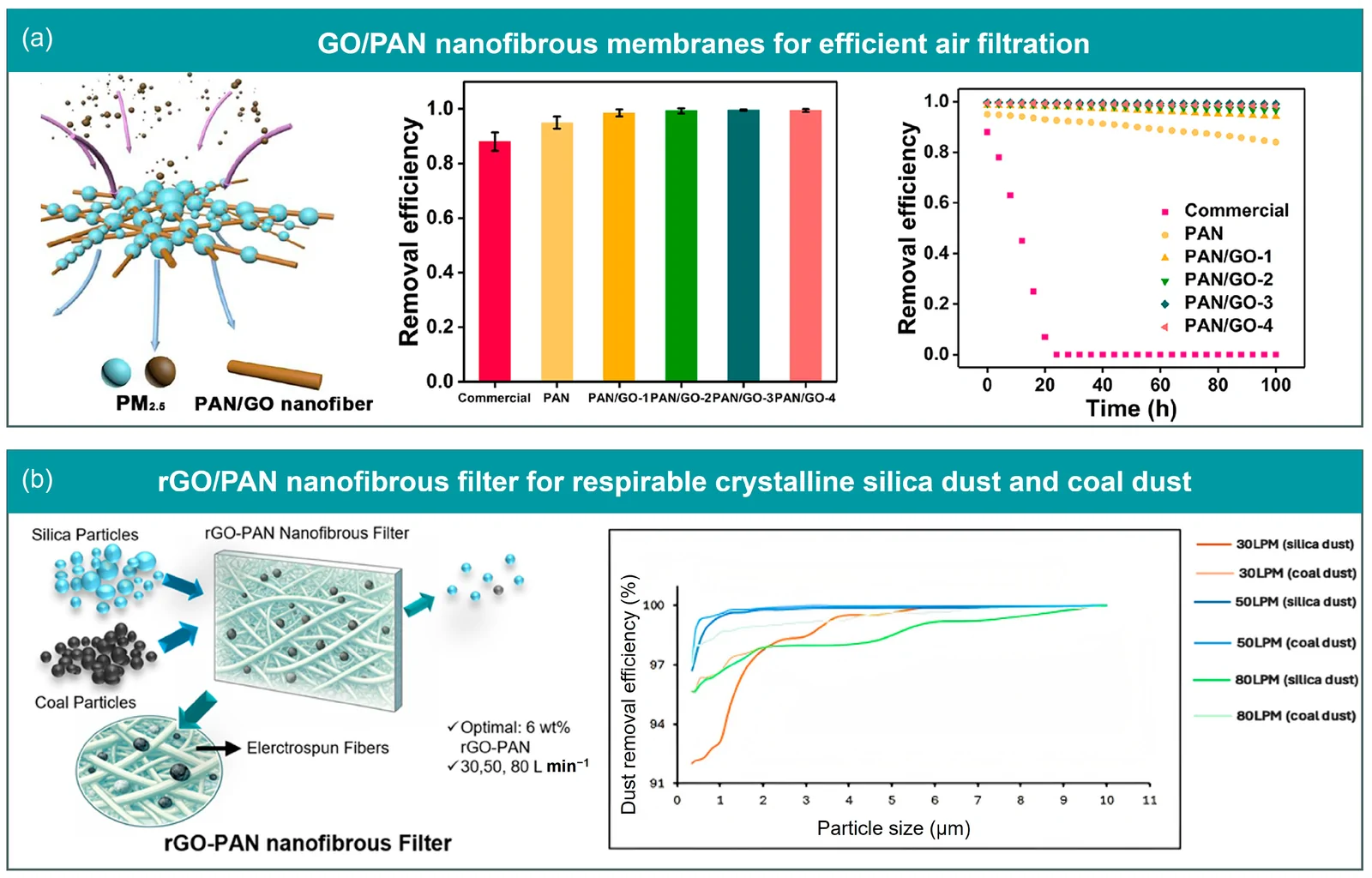
(**a**) Schematic of polyacrylonitrile/graphene oxide (PAN/GO) nanofibers, including PAN/GO-3 containing 0.05 wt% GO, comparison of PM2.5 removal efficiencies determined after 1000 s of testing, and removal performance over 100 h. (**b**) Schematic of reduced graphene oxide/polyacrylonitrile (rGO-PAN) filters and particle-size-dependent removal efficiencies for respirable crystalline silica and coal dust at an rGO loading of 6 wt%. PM2.5 denotes particulate matter with an aerodynamic diameter of 2.5 μm or less. LPM denotes litres per minute. Adapted from Ref. [48] [American Chemical Society], Copyright [2026].

Graphene-based additives provide a representative example. As shown in Figure 6, electrospun graphene oxide (GO)/PAN nanofibrous membranes have been reported to achieve PM_2.5_ removal efficiencies of approximately 99.6%, while retaining 99.1% efficiency after 100 h of operation [46]. In another GO/PAN system, the formation of a bead-on-string morphology generated larger inter-fiber voids and enabled a PM_2.5_ filtration efficiency of 99.97% at a pressure drop of only 8 Pa [47]. This enhancement is attributed to the GO-induced olive-like bead macrostructures, which not only expanded the inter-fiber voids to reduce airflow resistance but also provided a high specific surface area and additional capture sites for PM_2.5_ through hydrogen bonding and dipole–dipole interactions. These results suggest that nanofillers can improve filtration not only through enhanced surface interactions but also by reorganizing the fiber network. Thus, the role of an additive should be evaluated in terms of its structure-modifying effect, rather than simply its nominal loading.

This principle is particularly relevant to mining dust. A recent reduced graphene oxide (rGO)-PAN nanofibrous filter was evaluated against both respirable crystalline silica and coal dust as illustrated in Figure 6b, with the optimum rGO loading providing high removal efficiency for both particle types [48]. At a flow rate of 50 L min^−1^, the 6 wt% rGO-PAN filter achieved the highest efficiencies for both respirable crystalline silica (95.23%) and coal dust (94.21%), with complete removal of particles ≥ 8.032 μm. The excellent performance could be attributed to three mechanisms: (1) reduced fiber diameter (from 527 nm for pure PAN to 469 nm for 6 wt% rGO-PAN), increasing specific surface area and interception sites; (2) oxygen-containing functional groups on rGO (-OH, -COOH, C=O) forming dipole–dipole and hydrogen bonds with silanol groups on respirable crystalline silica and oxygenated groups on coal dust; and (3) a superior quality factor for the 6 wt% formulation at 50 and 80 L min^−1^, indicating an optimal efficiency–resistance balance. The use of realistic mining-related particles makes this work more representative of practical respiratory protection than studies based solely on standard laboratory aerosols. At the same time, the existence of an optimum rather than a continuously increasing benefit with rGO loading highlights an important limitation of nanoparticle incorporation: excessive loading can promote aggregation, alter fiber formation, and increase flow resistance.

Therefore, composite nanofibers should be regarded as structure–function platforms rather than simple additive-containing fibers. The most effective designs are those in which the secondary component simultaneously improves interactions between particles and fibers while preserving an open and mechanically stable transport network. This consideration naturally leads from compositional optimization toward more deliberate control of membrane architecture.

### 3.3. Hierarchical Architectures for Filtration-Transport Optimization

Structural engineering provides a further route to overcome the inherent trade-off between filtration efficiency and airflow resistance. Coaxial and side-by-side electrospinning can generate core-sheath and Janus fibers, while sequential electrospinning enables multilayer and gradient membranes [49,50,51]. These architectures allow different regions of a membrane to perform distinct functions rather than forcing a single fiber population to simultaneously satisfy all requirements.

**Figure 7:**
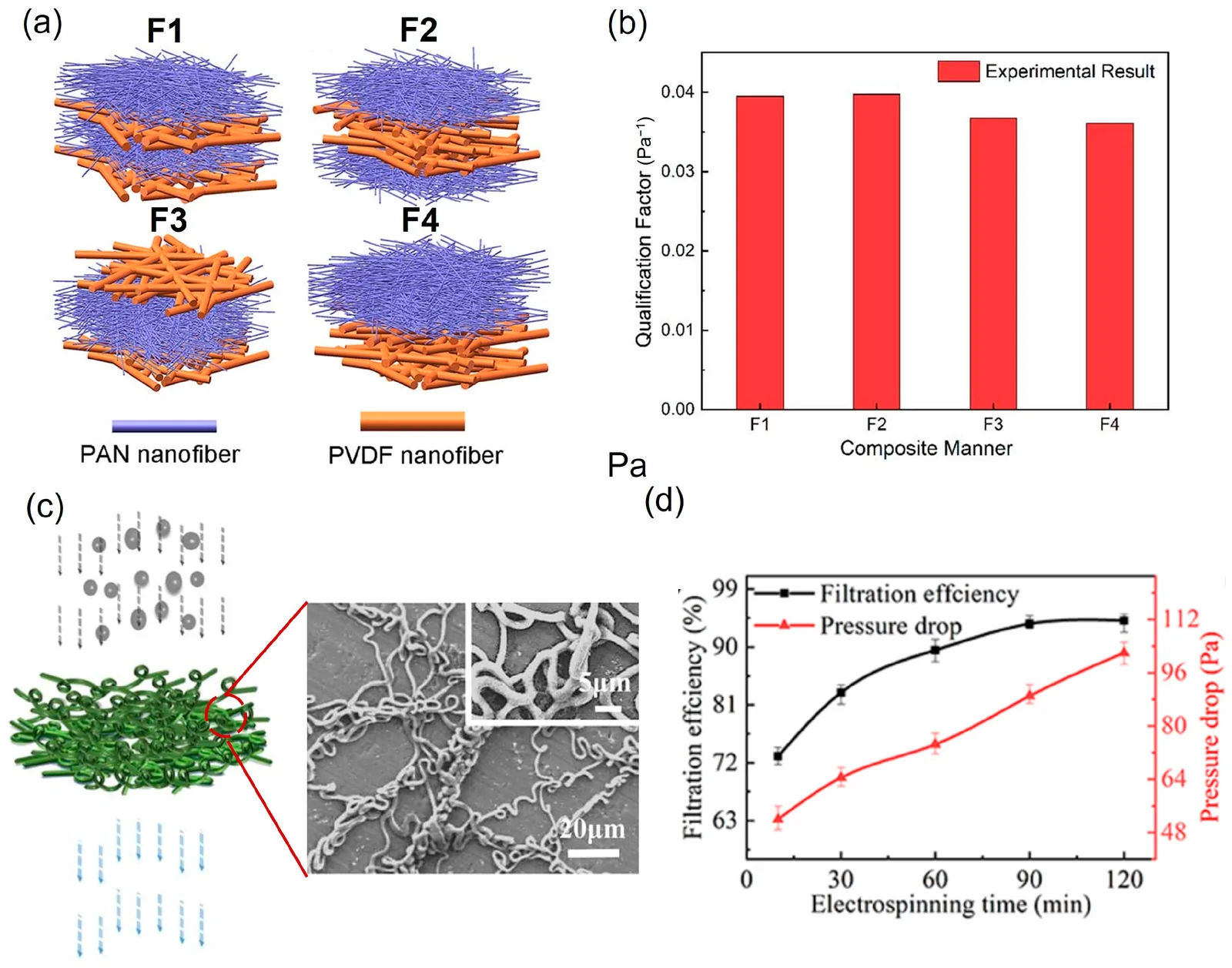
(**a**) Schematic diagrams of multi-layer nanofibrous membranes with different composite manners, (**b**) qualification factor of multi-layer nanofibrous membranes with different composite manners, adapted from Ref. [53] [IOP Publishing], Copyright [2021]. (**c**) Air filtration process filtration membrane with curled PVDF nanofiber and corresponding SEM image, (**d**) the filtration efficiency and pressure drop of PVDF nanofibrous membrane at different electrospinning time. Adapted from Ref. [54] [MDPI], Copyright [2021].

Bimodal nanofibrous membranes are particularly attractive because fine fibers provide efficient capture of small particles, whereas coarse fibers maintain larger flow channels and reduce excessive packing density [52]. More generally, hierarchical networks can distribute the filtration function across multiple length scales, thereby improving the efficiency–resistance balance. As shown in Figure 7a, a representative 3D membrane developed by Jiang et al. combined thin PAN nanofibers with thicker poly(vinylidene fluoride) (PVDF) fibers to form a more open supporting framework [53]. From Figure 7b, the resulting membrane achieved a maximum quality factor of 0.398 Pa^−1^, demonstrating that a three-dimensional fiber network can maintain efficient ultrafine-particle capture without relying solely on dense nanofiber packing. Similarly, three-dimensional curled PVDF nanofibrous membranes have been developed to increase the effective free volume of the filter (Figure 7c) [54]. From Figure 7d, the optimized structure achieved approximately 93.6% particle filtration efficiency with a pressure drop of 89 Pa, demonstrating the potential of three-dimensional architectures to improve the filtration and transport balance. These results indicate that structural optimization should extend beyond conventional parameters such as fiber diameter and basis weight toward control of fiber orientation, pore-size distribution, tortuosity, and three-dimensional connectivity.

From the perspective of mining protection, this shift is particularly important. A respirator must operate continuously under high dust loading, and a dense nanofiber layer that initially provides excellent filtration may rapidly accumulate particles and develop an unacceptable pressure drop. Hierarchical and multilayer architectures offer a potential solution by distributing particle capture spatially and maintaining more accessible airflow pathways. However, increasing architectural complexity also introduces additional interfaces and fabrication requirements. Therefore, structural sophistication is beneficial only when it produces a measurable improvement in the overall filtration–transport balance.

### 3.4. Humidity-Resistant and Moisture-Adaptive Membranes

**Figure 8:**
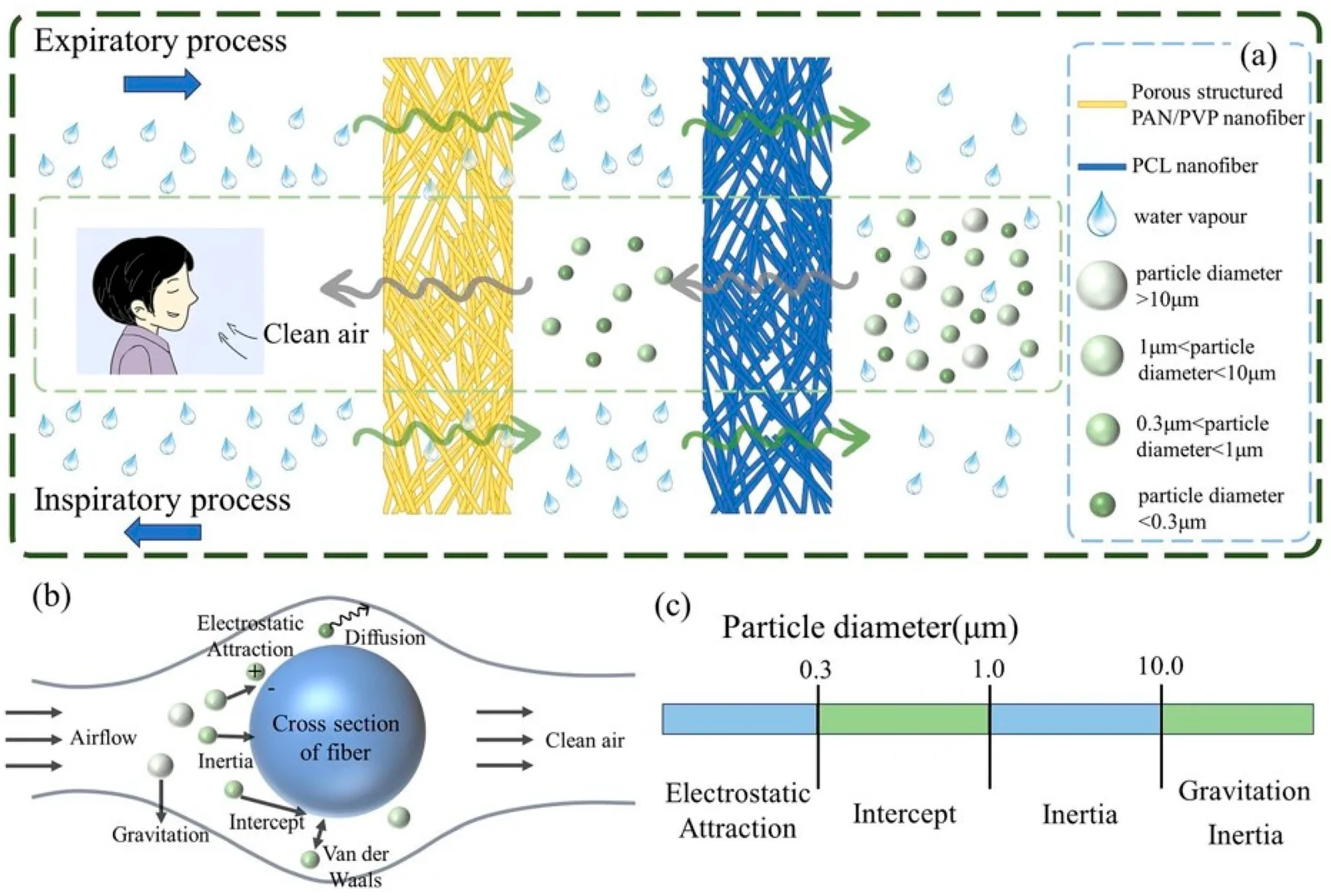
(**a**) Respiratory filtration mechanism diagram of Janus nanofiber membrane. Blue arrows represent inspiratory/expiratory airflow, green wavy arrows denote water-vapor transport, and gray arrows show particle movement. (**b**) mechanism of fiber capture on coal dust particles. The large hollow arrow indicates main airflow direction, and small arrows depict particle trajectories under different capture mechanism. (**c**) particle filtration mechanism with different particle sizes. Adapted from Ref. [56] [Elsevier], Copyright [2025].

The performance advantages of electrospun membranes can be substantially altered by humidity, making moisture resistance particularly important for underground mining environments. Water uptake can modify fiber morphology and particle adhesion, affect electrostatic charge retention, and increase pressure drop through changes in pore accessibility [55]. Hydrophobic modification and asymmetric wettability provide promising strategies for mitigating these effects. Instead of uniformly increasing hydrophobicity, Janus membranes can spatially separate water-repellent and water-transporting functions. Zhou et al. developed a porous Janus nanofiber membrane for personal dust protection, in which a hydrophobic PCL layer was sequentially electrospun onto a hydrophilic PAN/polyvinylpyrrolidone (PVP) layer to create asymmetric wettability and pore characteristics as illustrated in Figure 8 [56]. The hydrophobic outer layer suppresses external water penetration, whereas the hydrophilic inner layer facilitates the uptake and outward diffusion of water vapor from exhaled breath. The resulting wettability gradient promotes directional moisture transport while maintaining an open porous pathway for air flow. Under a high-intensity respiration rate of 85 L min^−1^, the membrane achieved a filtration efficiency of 99.98% with a pressure drop of 134.7 Pa, while maintaining efficient water-vapor transport. This design demonstrates how spatially differentiated wettability can simultaneously address moisture protection and breathability, offering a promising strategy for maintaining filtration performance under humid mining conditions.

More recently, gradient-wettability PAN membranes have been developed to combine moisture management with high particle-filtration performance. Such membranes maintain high filtration efficiency under elevated relative humidity while promoting directional water-vapor transport [57]. These studies suggest that humidity resistance should not simply be achieved by making the entire membrane hydrophobic. Instead, spatially controlled wettability can regulate water transport while preserving particle-capture functionality. For mining applications, this approach is particularly promising because high humidity, continuous respiration, and heavy dust loading occur simultaneously.

Overall, the evolution from single-polymer fibers to composite, hierarchical, and moisture-adaptive architectures reflects a broader transition from material optimization to engineering-level membrane design. Importantly, the best-performing membrane is not necessarily the one with the highest initial filtration efficiency, but the one that maintains a favorable balance among particle capture, airflow resistance, moisture tolerance, mechanical stability, and long-term durability. These engineering strategies establish the structural and material foundation for the next stage of development: integrating dust filtration with gas removal within a single electrospun membrane platform.

## 4. Toward Integrated Dust–Gas Protective Membranes

As discussed above, dust filtration alone cannot meet the requirements of respiratory protection in mining and metallurgical environments. Coal mining exposes workers to coal dust, respirable crystalline silica, and toxic gases such as CO, H_2_S, and NO_x_, while metallurgical processes can generate fine particulate matter together with SO_2_, VOCs, and other hazardous gases [58,59]. These pollutants coexist in complex atmospheres and can interact with each other during transport through a membrane [60]. Therefore, introducing gas-removal functions into the nanofibrous platform while preserving the low resistance and high particle-filtration efficiency is becoming a prevailing trend. A useful design principle is to separate the roles of the membrane components. The nanofiber network should provide mechanical support and efficient particle capture, while accessible functional phases should provide adsorption or catalytic sites for gas removal. This approach can be implemented through molecular adsorbents, porous fillers, MOFs, catalytic nanoparticles, or antimicrobial agents. More importantly, their spatial distribution should be controlled to avoid blocking the gas-accessible surface or excessively increasing airflow resistance. Since the preceding sections have established the material and structural strategies for effective particulate filtration, the following discussion focuses primarily on gas-removal functionalities and their integration with the established dust-filtration framework.

### 4.1. Gas-Adsorptive Functionality in Electrospun Nanofibers

Adsorption is one of the most direct approaches for introducing gas-removal capability into electrospun membranes. The basic strategy is to incorporate a porous or chemically active adsorbent into the nanofiber network while retaining sufficient open pathways for gas transport. Activated carbon, cyclodextrins, graphene derivatives, porous organic polymers, and other porous materials can provide additional interaction sites for gaseous molecules [8,61,62]. Their integration can be achieved by blending during electrospinning, surface deposition, electrospraying, or post-treatment.

Kadam et al. designed the PAN/β-cyclodextrin electrospun membrane for simultaneous particulate filtration and VOC adsorption [63]. The membrane achieved more than 95% particle-filtration efficiency with a pressure drop of 112 Pa, while the adsorption of formaldehyde and xylene increased by approximately 66% and 73%, respectively, compared with pristine PAN membranes. This result demonstrates an important advantage of nanofiber-based adsorptive membranes. The polymer network provides a lightweight filtration scaffold, while β-cyclodextrin introduces molecular recognition sites for VOC capture. However, the study also revealed that adding β-cyclodextrin reduced the fiber surface area, indicating that adsorption functionality does not necessarily increase with additive loading. This highlights the need to preserve both adsorbent accessibility and membrane permeability.

**Figure 9:**
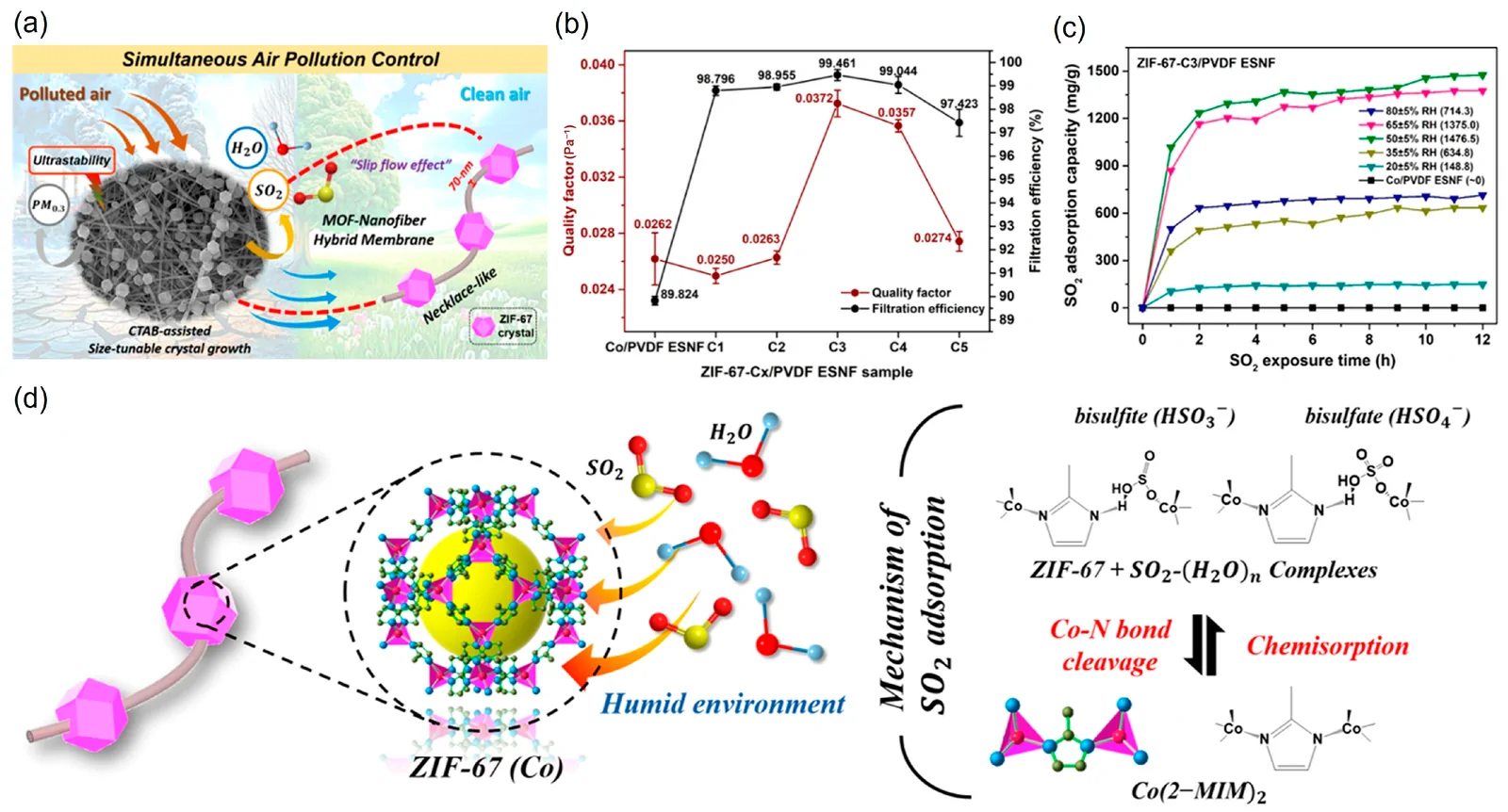
(**a**) Schematic illustration of a necklace-like ZIF-67/PVDF electrospun nanofiber membrane for simultaneous PM_0.3_ filtration and SO_2_ adsorption, (**b**) quality factor of the PM0.3 filtration efficiency of the Co/PVDF ESNF substrate and ultrastable ZIF-67-Cx/PVDF ESNF nanocomposite membranes, (**c**) effect of humidity on the dynamic SO_2_ adsorption performance of the ultrastable ZIF-67-C3/PVDF ESNF nanocomposite membrane, (**d**) schematic illustration of the SO_2_ adsorption mechanism. Adapted from Ref. [65] [American Chemical Society], Copyright [2024].

For mining and metallurgical applications, the same principle can be extended to activated carbon and other high-surface-area sorbents targeting SO_2_, VOCs, and reduced sulfur compounds. For example, electrospun carbon nanofiber-supported Fe sorbents have recently been developed for H_2_S removal from high-temperature coal gas, demonstrating the potential of electrospun porous carbon frameworks for treating sulfur-containing gases under harsh industrial conditions [64]. Meanwhile, Kim and co-workers developed a necklace-like ZIF-67/PVDF electrospun nanofiber membrane for simultaneous PM_0.3_ filtration and SO_2_ adsorption as shown in Figure 9a [65]. The ZIF-67 particles were selectively distributed along the PVDF nanofibers, providing accessible microporous sites for SO_2_ adsorption while preserving the interconnected nanofibrous network for particle capture. From Figure 9b,c, the optimized membrane achieved a PM_0.3_ filtration efficiency of 99.461% and an SO_2_ adsorption capacity of 1476.5 mg g^−1^, while maintaining stable performance under different humidity conditions. As illustrated in Figure 9d, the nanofibrous network captures PM_0.3_ via interception, inertial impaction, and Brownian diffusion, while its 70 nm fiber diameter provides a slip effect that reduces pressure drop. Meanwhile, the ZIF-67 crystals enhance filtration through their large surface area, microporous structure, and unsaturated Co^2+^ sites, which promote electrostatic interactions with fine particles. This work provides a particularly relevant example of dust-gas integration because the same membrane architecture simultaneously addresses fine-particle filtration and a chemically distinct gaseous pollutant. More importantly, the necklace-like configuration demonstrates that the spatial accessibility of the adsorbent is as important as its intrinsic adsorption capacity, which is critical for designing low-resistance protective membranes for complex industrial atmospheres.

It should be noted that the challenge becomes more complex when particulate matter is present simultaneously. Dust deposition can cover adsorption sites and increase gas-phase mass-transfer resistance. Thus, adsorptive membranes should be designed so that the gas-active phase remains accessible after prolonged dust exposure. A layered configuration can be advantageous in this respect, with an upstream nanofibrous layer providing particle interception and a downstream adsorptive layer maintaining access to gas-binding sites.

### 4.2. Selective Gas Capture in Electrospun Nanofibrous Membranes

Unlike particulate filtration, gas removal requires not only sufficient adsorption capacity but also selective interaction with target molecules in complex atmospheres. This requirement is particularly important in mining and metallurgical environments, where numerous toxic gasses can coexist with large concentrations of dust and moisture. A highly porous adsorbent may provide a large number of adsorption sites, but high surface area alone does not guarantee effective removal of a specific toxic gas. Pore size, surface chemistry, molecular affinity, and resistance to competitive adsorption all influence the selectivity and practical efficiency of gas capture [66,67,68]. Therefore, selective gas removal requires adsorbents whose pore structures and surface chemistry can be deliberately tailored to the target pollutant.

Among emerging porous adsorbents, MOFs are particularly attractive because their pore size, topology, and surface chemistry can be systematically tuned through the selection of metal nodes and organic ligands [69]. This structural tunability allows MOFs to provide stronger interactions with specific gas molecules than conventional activated carbon, while their high internal surface areas offer abundant adsorption sites. These characteristics are especially relevant for the selective capture of sulfur-containing and polar gases such as H_2_S, SO_2_, and NH_3_ [70,71,72]. However, MOFs are commonly obtained as fine powders, which limits their direct use in flexible respiratory filters because of poor mechanical integrity and possible particle loss. Integrating MOFs with electrospun nanofibers therefore provides a practical route to combine molecular selectivity with membrane processability and particle filtration.

**Figure 10:**
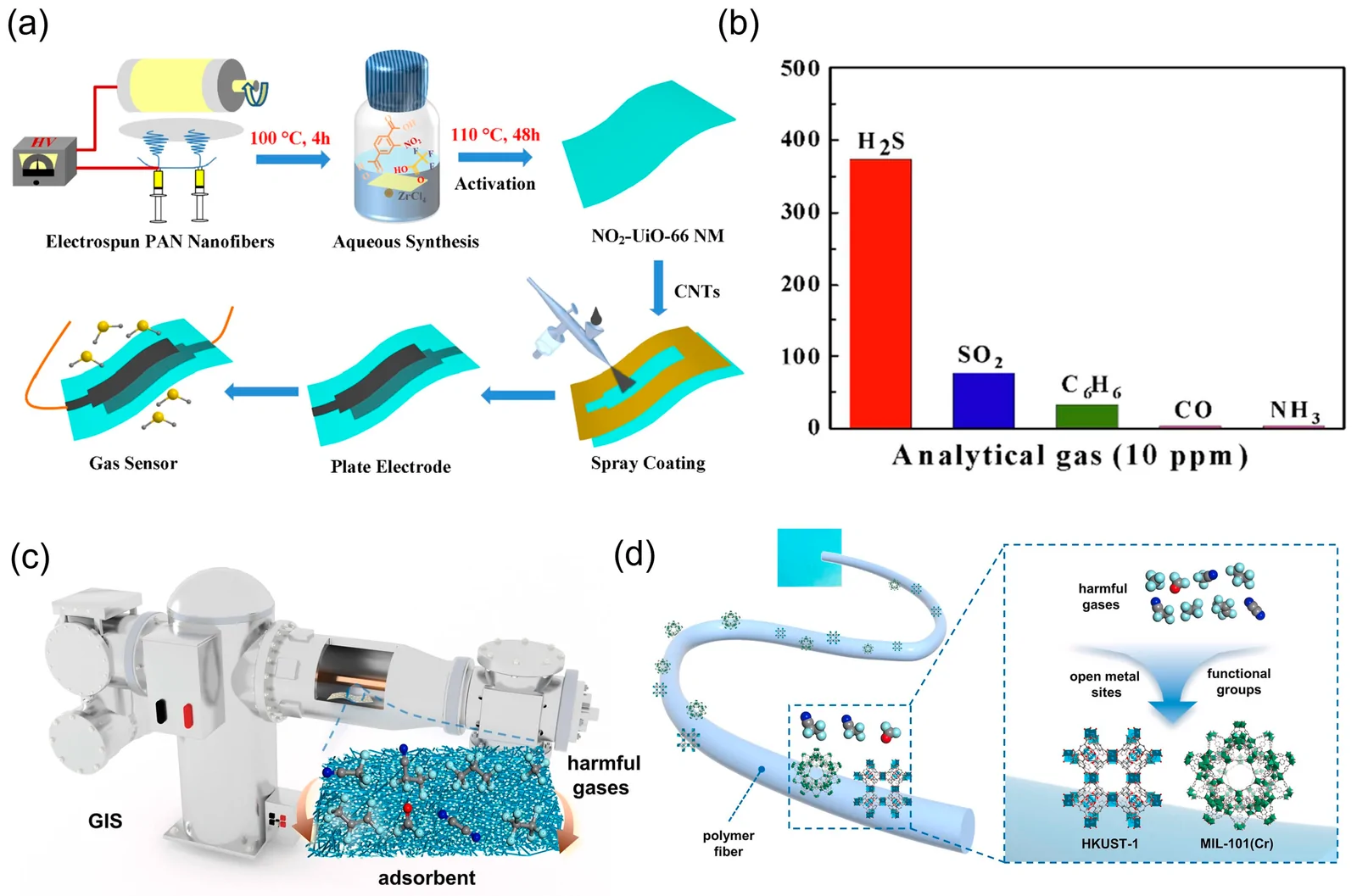
(**a**) The schematic illustration of fabricating process for capacitive gas sensor based on NO_2_-UiO-66@PAN NM, (**b**) selectivity of the NO_2_-UiO-66 NM sensor to H_2_S vs. SO_2_, C_6_H_6_, CO, NH_3_ at 10 ppm, Adapted from Ref. [73] [Elsevier], Copyright [2022]. (**c**) Schematic illustration of the adsorbent used in GIE, (**d**) proposed capture mechanism of harmful gases. Adapted from Ref. [74] [Elsevier], Copyright [2024].

Selective gas capture requires more than a high adsorption capacity. The adsorbent must preferentially interact with the target molecule in the presence of competing gases. Zhang et al. reported a NO_2_-UiO-66/electrospun PAN nanofiber membrane, in which NO_2_-UiO-66 was grown uniformly on the fiber surface to provide accessible and chemically active adsorption sites as illustrated in Figure 10a [73]. The resulting membrane showed a pronounced response toward H_2_S compared with SO_2_, benzene, CO, and NH_3_, with a detection limit as low as 10 ppb (Figure 10b). This result demonstrates that the integration of MOFs with electrospun nanofibers can provide not only high gas accessibility but also molecular-level selectivity, which is particularly important for distinguishing target toxic gases in complex industrial atmospheres.

A further example was reported by Xiao et al., who developed a PAN@MOFs composite nanofiber membrane for the selective removal of C_4_F_7_N and its decomposition products in gas-insulated equipment (GIE) (Figure 10c) [74]. The MOF phase was directly integrated with the electrospun PAN nanofiber framework, providing tailored microporous environments for molecular recognition. The selective adsorption originated from the stronger host–guest interactions between the MOF pore environment and the target molecules, together with favorable molecular diffusion within the pore channels (Figure 10d). This work highlights that MOFs can enhance gas selectivity not simply through high surface area, but through the precise matching of pore structure, surface chemistry, and molecular interactions. Such molecular-level design is particularly valuable for mining and metallurgical environments, where target toxic gases coexist with abundant background gases and water vapor.

For mining and metallurgical protection, however, selective gas capture must be further coupled with particulate filtration. It could be required to retain the molecular recognition capability of the MOF while incorporating sufficient nanofibrous structure for efficient dust removal. This provides careful control over MOF loading, crystal distribution, and exposure of adsorption sites. Excessive embedding of MOF crystals inside polymer fibers can limit gas diffusion, whereas excessive surface loading may compromise fiber integrity and increase airflow resistance. Therefore, the key design objective may be to create an electrospun membrane in which the nanofibrous network provides efficient particle interception while the exposed MOF phase selectively captures specific toxic gases.

### 4.3. Catalytic Gas Removal and Self-Regenerating Protection

Adsorption alone is limited by the gradual saturation of active sites [75,76]. This limitation is particularly important for mining and metallurgical applications because protective membranes may be exposed continuously to toxic gases. Catalytic degradation provides a complementary strategy in which gaseous pollutants are converted into less harmful products rather than simply stored within the membrane [77].

**Figure 11:**
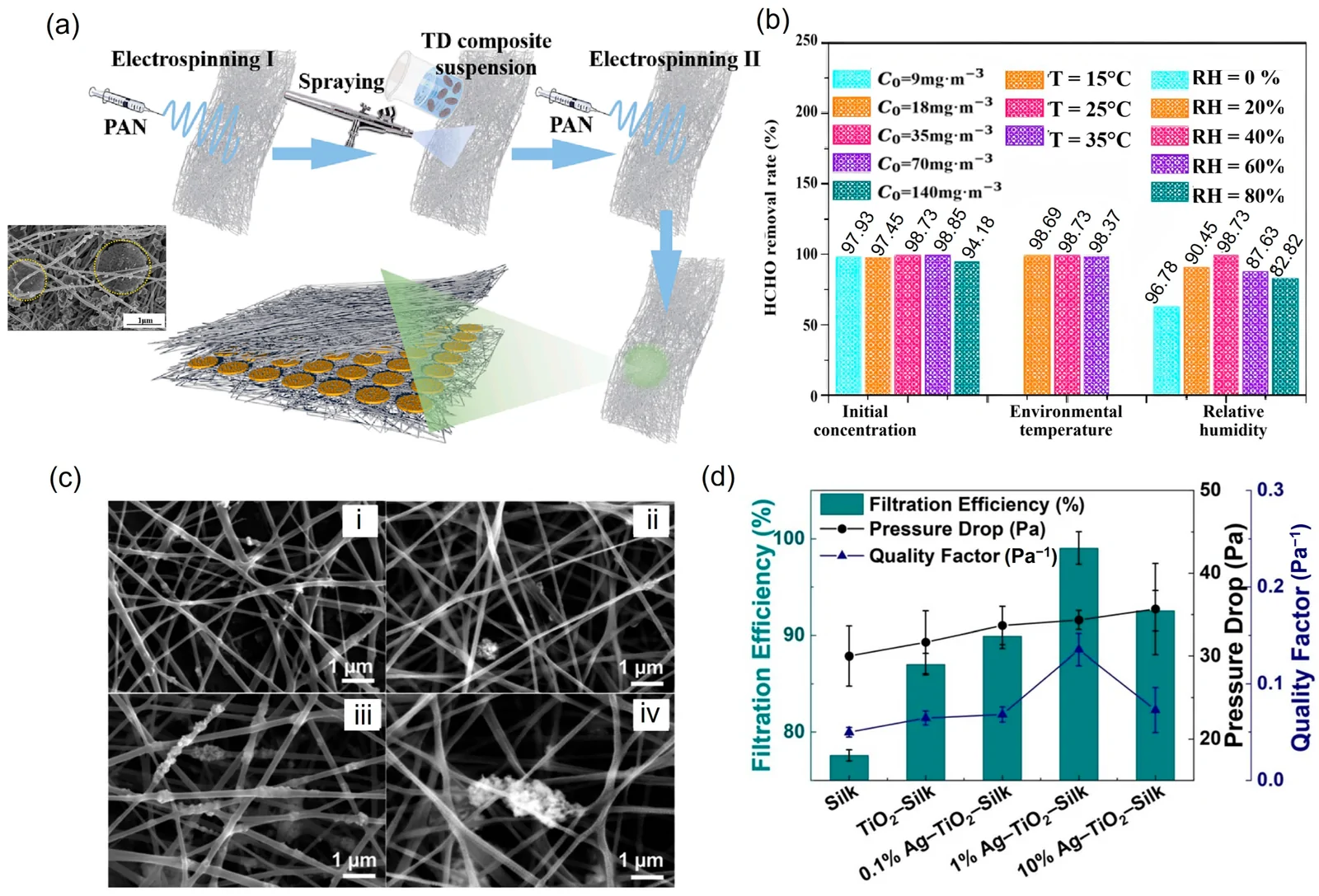
(**a**) Synthesis process of TiO2@diatomite/PAN composite nanofiber membrane, the inset is the SEM images of the 0.8 TiO2@diatomite/PAN, (**b**) HCHO removal rate of different environmental factors, adapted from Ref. [79] [Elsevier], Copyright [2023]. (**c**) SEM images of (**i**) TiO_2_-silk, (**ii**) 0.1%Ag-TiO_2_-silk, (**iii**) 1%Ag-TiO_2_-silk, and (**iv**) 10%Ag-TiO_2_-silk, (**d**) PM_2.5_ filtration efficiency, pressure drop and quality factor of silk, TiO_2_-silk, and Ag-TiO_2_-silk nanofibers at initial 20 min filtration. Adapted from Ref. [80] [RSC], Copyright [2023].

Photocatalytic nanoparticles such as TiO_2_ and ZnO can be incorporated into electrospun nanofibers to provide reactive sites for gas degradation [78]. Under irradiation, photogenerated electrons and holes generate reactive oxygen species that oxidize adsorbed pollutants. The nanofibrous network provides a porous pathway for gas diffusion and supports the catalyst. For example, a TiO_2_@diatomite/PAN composite nanofiber membrane was fabricated by electrospinning-spraying for photocatalytic removal of gaseous formaldehyde (Figure 11a) [79]. The diatomite-supported TiO_2_ enhanced pollutant adsorption while providing accessible photocatalytic sites, and the authors further examined the effects of temperature, humidity, and initial HCHO concentration on the degradation process (Figure 11b). This work highlights the importance of coupling adsorption and photocatalysis within an electrospun structure, although its dependence on irradiation remains a limitation for poorly illuminated underground environments.

A critical distinction is required when interpreting such results. A decrease in gas-phase concentration does not by itself prove degradation, since a pollutant may either be irreversibly mineralized to CO_2_ and H_2_O or merely adsorbed and retained within the polymer matrix and later desorbed. True mineralization should be verified from the end products, namely a measured increase in CO_2_ with a closed carbon balance, the absence of concentration rebound after irradiation, and controls that separate dark adsorption from photocatalytic conversion. Yuan et al. investigated HCHO removal using a TiO_2_@diatomite/PAN membrane and used in situ diffuse reflectance infrared Fourier transform spectroscopy (DRIFTS) to examine reaction intermediates and propose a degradation pathway [79]. These findings support photocatalytic HCHO removal, but they are not presented here as evidence of near-complete mineralization. Such a conclusion requires a quantified CO_2_ yield relative to the HCHO carbon input or a carbon balance. Partial oxidation of VOCs can instead yield intermediate by-products such as aldehydes, carboxylic acids, or CO, which may be as harmful as the parent compound and should be identified rather than assumed absent.

The light requirement of these systems is a further limitation. Anatase TiO_2_ has a bandgap of approximately 3.2 eV and is activated mainly by UV light, while Ag doping extends some activity into the visible range but remains limited. The practical difference is considerable. Under UV irradiation, the TiO_2_@diatomite/PAN membrane removed 97.19% of HCHO within 45 min, whereas under actual sunlight over 6 h removal fell to 78.83%. Because underground mines receive essentially no natural light and only limited artificial illumination, photocatalytic membranes may operate well below their laboratory efficiency or may not be activated at all. Design should therefore pair photocatalysis with an adsorptive phase that retains pollutants during dark periods, or adopt visible-light-responsive catalysts with an integrated low-energy light source.

Therefore, catalytic integration should complement rather than replace adsorption. An adsorptive component can concentrate gas molecules near catalytic sites, while continuous catalytic conversion can reduce adsorbent saturation. As shown in Figure 11c, an electrospun Ag-doped TiO_2_/silk nanofiber membrane, for example, combined PM_2.5_ filtration, VOC degradation, and antibacterial activity, achieving 99.04 ± 1.70% PM_2.5_ removal with a pressure drop of 34.3 Pa (Figure 11d) [80]. Such integration demonstrates the potential of combining particle capture, gas conversion, and additional protection within one fibrous membrane.

From an engineering perspective, the spatial arrangement of catalytic particles is again critical. Excessive incorporation within dense fibers may shield active sites from gas molecules and light, whereas surface immobilization can improve accessibility but increase the risk of particle loss. A Janus or multilayer configuration may therefore be advantageous, with a particle-filtering layer positioned upstream and an exposed catalytic layer located downstream. Such an arrangement can reduce dust fouling of catalytic sites while maintaining gas accessibility.

### 4.4. Antimicrobial Protection for Humid and Dust-Loaded Environments

In addition to dust and toxic gases, prolonged exposure to humid and dust-loaded environments creates another challenge for reusable protective membranes. Moisture and deposited organic matter can provide favorable conditions for microbial attachment and growth [81]. This issue is especially relevant to respirators used repeatedly in underground mining and industrial environments. Antimicrobial functionality can therefore extend the protective role of electrospun membranes beyond pollutant removal.

**Figure 12:**
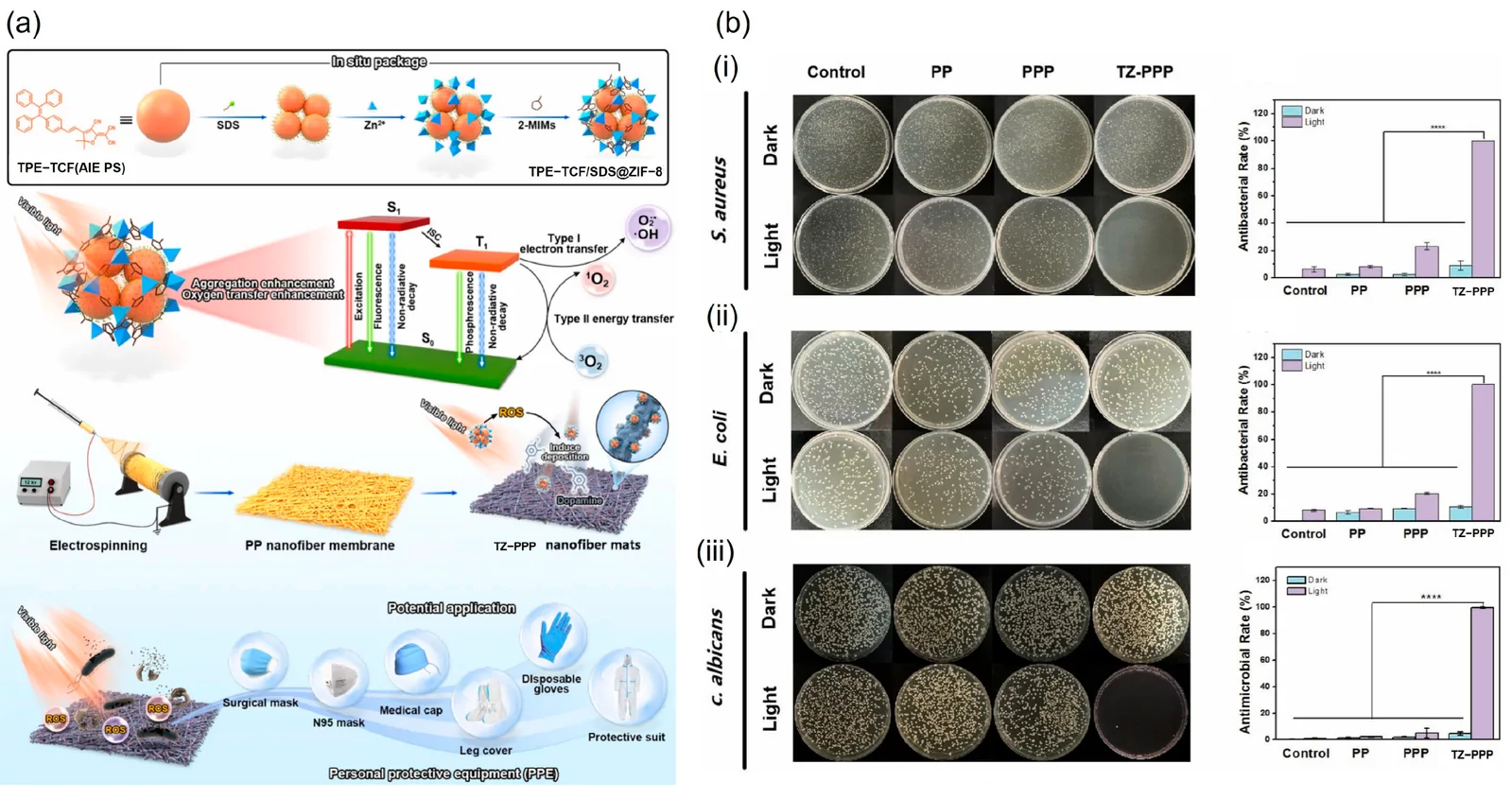
(**a**) Schematic preparation of (E)-2-(3-cyano-5,5-dimethyl- 4-(4-(1,2,2-triphenylvinyl)-styryl)furan-2(5H)-ylidene)malononitrile(TPE-TCF)/Sodium dodecyl sulfate(SDS)@ZIF-8 NPs and TPE-TCF/SDS@ZIF-8+ Polyacrylonitrile/Polyvinylpyrrolidone (TZ-PPP) nanofiber mats, as well as antimicrobial mechanisms and applications, (**b**) (**i**) representative plate images of *S. aureus* on LB agar plates, and histogram of the relative antibacterial rate (%) corresponding to treatments, (**ii**) representative plate images of *E. coli* a on Lysogeny broth (LB) agar plates and histogram of the relative antibacterial rate (%) corresponding to treatments. (**iii**) Representative plate images of *C. albicans* on potato dextrose agar plates, and histogram of the relative antibacterial rate (%) corresponding to treatments. The symbol **** indicates a statistically significant difference with *p* < 0.0001. Adapted from Ref. [83] [Elsevier], Copyright [2026].

Recent work has demonstrated that electrospun nanofibers can serve as platforms for integrating antibacterial agents while maintaining their porous structure. For example, PAN nanofibers doped with ZnO and Cu nanoparticles and coated with an antibacterial chitosan layer achieved a 99.9% bactericidal rate against E. coli and S. aureus while retaining their filtration performance after washing [82]. More recently, an aggregation-induced emission (AIE) photosensitizer was encapsulated in ZIF-8 nanoparticles and assembled onto PAN/PVP electrospun nanofiber mats as shown in Figure 12a [83]. Under visible light, the resulting membrane achieved more than 99.9% bacterial removal within 10 min and 99.0% fungal removal within 40 min through photodynamic generation of reactive oxygen species (Figure 12b). Like the photocatalytic gas-removal systems above, this photodynamic route requires visible-light activation and is therefore subject to the same illumination constraints in underground environments. These studies provide an interesting extension of the dust-gas protection concept. The same nanofibrous platform can potentially integrate particle filtration, gas adsorption or catalytic conversion, and antimicrobial activity. However, adding multiple functions also increases the risk of pore blockage, pressure-drop growth, active-site shielding, and material instability. Therefore, multifunctionality should not be pursued by continuously increasing the number of functional components. Instead, each function should be assigned to an appropriate location within the membrane.

Overall, the integration of gas-removal functionalities provides a critical pathway toward multifunctional electrospun protective membranes. Adsorption-based materials offer high pollutant-capture capacity, selective porous adsorbents enable target-specific gas removal, while photocatalytic components provide the possibility of continuous pollutant degradation and reduced saturation. Antimicrobial functionalities can further improve the durability and safety of membranes under humid and dust-loaded conditions. However, these functions should not be considered independently, as gas accessibility, humidity, dust loading, pressure drop, and long-term stability are strongly coupled within the membrane. Future designs should therefore focus on spatially and functionally coordinated architectures that maintain efficient particulate filtration while providing accessible, selective, and durable gas-removal sites. Such integration represents an important step toward next-generation protective membranes for complex mining and metallurgical environments.

## 5. Challenges and Future Perspectives

Despite the progress reviewed above, several challenges remain for the practical application of electrospun membranes in mining environments. These challenges span material design, environmental adaptability, and system integration. They also point toward future research directions. Here, Table 1 consolidates the reported performance and test conditions of representative electrospun membranes. It can be inferred that the electrospun nanofibrous membranes provide efficient dust filtration with high efficiencies from 93.6 to 99.6%, confirming that fine fibers achieve high capture efficiency. Moreover, the same platform supports integrated dust-gas protection when gas-active phases such as MOFs or cyclodextrin are placed at surface-accessible sites. However, the reported values are obtained under widely differing airflow, particle-size, and humidity conditions, and gas performance is generally given as a relative reduction rather than a capacity measured under dust-loaded, humid conditions. These inconsistencies frame the three challenges discussed below.

### 5.1. Spatial Organization of Functional Components

The first challenge is the spatial organization of dust-filtration and gas-removal functions within a single membrane. Current designs often combine functional components through blending, coating, or layering [25,84,85]. Each method has limitations. Blending can bury adsorption sites inside polymer fibers. Coating may block pores or detach under airflow. Layering introduces interfaces that may delaminate or restrict gas transport.

Future work should focus on controlled placement of functional phases. Electrospraying, sequential electrospinning, and surface growth techniques offer routes to achieve this control. Electrospraying enables the deposition of functional nanoparticles or adsorbents onto the surface of electrospun fibers without embedding them within the polymer matrix [86]. Sequential electrospinning allows the construction of multilayer or gradient architectures by successively depositing different fiber layers [87]. Surface growth techniques, particularly in situ growth of metal–organic frameworks (MOFs), produce chemically anchored functional coatings on fiber surfaces through coordination or nucleation processes [88]. However, the relationship between functional layer thickness, pore structure, and long-term pressure-drop evolution remains poorly understood. Systematic studies are needed to determine how dust loading affects gas accessibility in layered configurations. The goal is to create membranes in which the dust-capturing layer reduces particle penetration without blocking gas transport to the downstream adsorptive or catalytic layer.

Another open question is the trade-off between functional integration and mechanical durability. Adding MOFs, catalysts, or antimicrobial agents can alter fiber flexibility and tensile strength. Under repeated handling or high airflow, particle shedding or structural failure may occur. Future research should therefore evaluate mechanical stability alongside filtration and adsorption performance. Standardized testing protocols for integrated membranes would help compare different designs and guide optimization.

Quantitative data are still scarce but indicate the measurements that such protocols should include. The porous Janus membrane retained tensile strengths of 1.61 to 6.08 MPa and elongations at break of 138 to 184% after layer assembly [56], and the TiO_2_@diatomite/PAN membrane retained a tensile strength above 6.10 MPa [79], showing that the nanofiber scaffold can remain flexible after particle loading. Durability should nevertheless be measured rather than assumed. Repeated bending that mimics breathing and prolonged exposure to the working airflow should be followed by gravimetric measurement of mass loss and inspection for particle shedding, while tensile strength before and after cycling gives a direct measure of structural retention.

Chemical safety is a related concern and is even less documented. The incorporated MOFs and catalysts introduce heavy metals such as Co [65], Cu [74], Zn [83], and Ag [80], together with biocidal agents. Contact with sweat, breath condensate, and the acidic water formed when SO_2_, H_2_S, or CO_2_ dissolve can promote their release. Leaching should therefore be evaluated by immersion in simulated sweat or acidic media, with the extracted metals quantified by ICP-MS or ICP-OES and the biocides by chromatography, to ensure that the membrane does not convert airborne hazards into dermal or ingestion exposure.

### 5.2. Environmental Adaptability and Humidity Resistance

The second challenge is environmental adaptability. Mining atmospheres vary widely in humidity, temperature, and contaminant composition. A membrane that performs well under dry laboratory conditions may fail in a humid mine. Moisture affects particle adhesion, electrostatic charge, and gas adsorption. It can also promote microbial growth on reusable membranes.

Moisture-resistant designs have been demonstrated using hydrophobic polymers, Janus structures, and gradient wettability [56,89,90]. However, these approaches have mostly been evaluated for particle filtration alone. Their compatibility with gas-removal functionalities is less explored. For example, a hydrophobic surface may repel water but also reduce the accessibility of polar adsorption sites for gases such as NH_3_ or SO_2_ [91]. Future research should investigate how wettability gradients influence both moisture transport and gas adsorption simultaneously.

Another aspect is the stability of functional components under humid conditions. Many MOFs and metal oxides degrade or lose capacity upon water exposure. Surface coatings or hydrophobic modification can protect moisture-sensitive adsorbents, but they may also introduce additional mass-transfer resistance [92,93]. The development of water-stable adsorbents that maintain selectivity under high humidity is therefore a priority. Alternatively, catalytic components that convert pollutants under humid conditions could reduce the reliance on adsorption alone.

Retention of the active structure should be verified directly. X-ray diffraction before and after exposure to humidity or acidic gas allows the crystallinity of a MOF to be assessed from the persistence of its characteristic peaks. The ZIF-67/PVDF membrane illustrates this practice. Its crystalline structure was confirmed by XRD after SO_2_ exposure and humidity cycling, consistent with stable performance over 20 to 80% RH [65]. XPS and FTIR can identify changes in the metal coordination environment, and TGA provides a complementary measure of stability. Repeated-use tests are equally informative. The TiO_2_@diatomite/PAN membrane was also reported to exhibit reusability and structural stability during cycling [79], and the Ag-TiO_2_/silk membrane retained 86% PM removal after ten cycles [80]. Reporting such retention together with the analysis conditions would allow different designs to be compared on a quantitative basis.

Temperature variation is also relevant. Mining environments can be hot and poorly ventilated. Elevated temperatures reduce physisorption capacity and may affect the stability of electrospun fibers [94]. Future studies should examine the combined effects of temperature, humidity, and dust loading on membrane performance. Such studies would provide a more realistic basis for design than ambient-condition tests.

### 5.3. Integration of Multiple Protective Functions

The third challenge is the integration of multiple protective functions within a single membrane platform. Dust filtration, gas adsorption, catalytic degradation, and antimicrobial activity each place different demands on the membrane. Filtration requires open pores and mechanical strength. Adsorption requires exposed active sites and short diffusion paths. Catalysis requires light or thermal activation and stable catalyst support. Antimicrobial functionality requires contact-killing or release of biocidal agents.

Combining these functions without adverse interactions is difficult. For example, catalytic nanoparticles may agglomerate and block pores [95]. Antimicrobial agents may leach or lose activity over time [96]. MOFs may degrade during photocatalytic reactions [97]. Excessive functionalization increases fabrication complexity and reduces reproducibility [15]. Therefore, future designs should prioritize functional synergy over additive complexity.

One promising strategy is to assign different functions to different layers or regions of the membrane. An upstream layer could handle dust capture. A middle layer could provide gas adsorption or catalysis. A downstream layer could offer antimicrobial protection. This spatial separation reduces interference between functions. It also inevitably increases membrane thickness and may raise pressure drop. The optimal layer thickness and ordering must be determined for each application. Another strategy is to develop multifunctional materials that combine two or more functions intrinsically. For example, certain MOFs exhibit both gas adsorption and photocatalytic activity [98]. Some metal oxides provide both catalytic degradation and antimicrobial effects [99]. Such materials could reduce the number of components needed and simplify membrane fabrication. However, their integration into electrospun fibers without loss of functionality remains challenging.

Finally, the evaluation of integrated membranes must move beyond single-pollutant tests. Mining atmospheres contain mixtures of particles, gases, and moisture. Performance metrics should include simultaneous dust loading, gas breakthrough, pressure-drop evolution, and humidity tolerance. Long-term stability and regeneration capacity are also essential for practical use. Standardized protocols for such multicomponent testing are currently lacking and would accelerate the development of next-generation protective membranes.

To address this gap, we propose a minimum five-stage evaluation matrix that can be applied consistently to integrated membranes.

(i) Dry testing. Baseline filtration efficiency and pressure drop are measured at a defined particle size and face velocity under dry conditions, establishing the reference performance of the membrane.
(ii) Gas breakthrough assessment. The target gas is introduced at a fixed concentration and flow rate, and the breakthrough curve is recorded to determine the breakthrough time and adsorption capacity.
(iii) Humidity competition evaluation. The gas measurement is repeated at specified relative humidity levels to quantify how water vapor competes with the target gas for adsorption sites.
(iv) Dust–gas co-exposure testing. The membrane is exposed simultaneously to particles and the target gas under humid conditions, revealing whether dust loading alters gas access and whether the combined performance differs from the single-function results.
(v) Regeneration and flexural cycling evaluation. After repeated regeneration and bending cycles, filtration efficiency, gas capacity, mass loss, and particle shedding are re-measured to assess durability and functional retention.

The matrix progresses from baseline performance to realistic co-exposure and long-term use and provides a common set of metrics that would allow different architectures to be compared on the same basis.

## 6. Conclusions

This review has examined the design of electrospun nanofibrous membranes for integrated dust–gas protection in mining environments. Particulate filtration and gas removal operate on different principles. Particle capture is governed by aerodynamic interactions and fiber geometry. Gas removal depends on molecular interactions with active sites. An integrated membrane must combine both functions within a single porous structure. The analysis shows that gas-removal performance is determined not only by the intrinsic capacity of the adsorbent but also by its spatial accessibility within the fiber network. High loading does not ensure high efficiency if active sites are buried or diffusion paths are obstructed. Surface immobilization, electrospraying, and hierarchical layering are more effective than uniform blending for preserving accessibility. Dust filtration and gas removal are coupled through the membrane architecture. Dust deposition blocks pores and covers adsorption sites. Humidity modifies particle adhesion, reduces electrostatic charge, and competes for gas-binding sites. These interactions mean that integrated membranes cannot be designed by optimizing each function independently. The transition from single-polymer membranes to composite, hierarchical, and multifunctional designs reflects a broader shift in thinking. The best-performing membranes are not those with the highest value of a single property. They are those that maintain a stable balance among particle capture, gas removal, airflow resistance, moisture tolerance, and mechanical durability under realistic mining conditions. Future progress will depend on addressing the challenges of spatial organization, environmental adaptability, and multifunctional integration. Controlled placement of functional components, moisture-resistant architectures, and synergistic material combinations are key directions. Standardized testing under multicomponent and realistic exposure conditions is essential to translate laboratory advances into practical respiratory protection for miners.

## Figures and Tables

**Table 1:** Comparative summary of the reported filtration/gas-removal performance of electrospun nanofibrous membranes.

Ref.	Membrane Architecture	Polymer & Functional Components	Target Contaminant(s)	Filtration Efficiency	Particle Size	Airflow Rate/Face Velocity	Pressure Drop	Quality Factors	Gas-Removal Efficiency (%)/Gas-Adsorption Capacity (mg g^−1^)	Relative Humidity
[41]	Single-layer	PAN nanofiber network	NaCl solid particles	94.9%	0.3–0.4 μm	32.4 L/min (face velocity 0.15 m/s)	106 Pa (at 0.15 m/s)	NR	NR	NR
[42]	Blend	PAN/LiCl	NaCl aerosol particles	99.54%	0.3–10 µm	32 L/min	142.8 Pa	0.039 Pa^−1^	NR	30% (25 °C)
[46]	Blend	PAN/GO	PM_2.5_	99.6%	<2.5 μm	0.2 m/s	117 Pa	0.0472 Pa^−1^	NR	NR
[48]	Blend	rGO-PAN	Respirable crystalline silica dust and coal dust	~97%	0.3–10 μm	50 L/min	50 Pa	>0.03 Pa^−1^	NR	NR
[54]	Single-layer	PVDF with self-supporting 3D curled structure	NaCl aerosol particles	93.6%	0.3 μm	80 L/min;	89.0 Pa	0.0274 Pa^−1^	NR	30% (25 °C)
[33]	Multilayer composite	PVA@MOFs composite	PM_2.5_ (NaCl challenge aerosol)	97%	<2.5 μm	32 L/min	135 Pa	0.0289 Pa^−1^	~60% for NH_3_; 35% for H_2_S (Gas-removal efficiency)	NR
[63]	Functionalized single layer	PAN/β-cyclodextrin (β-CD)	KCl aerosols	>95%	0.3–5 μm	20 L/min (face velocity 0.06 m/s)	92 Pa	0.027 Pa^−1^	66% for HCHO; 73% for xylene (Gas-removal efficiency)	50 ± 2% (20 ± 2 °C)
[65]	Composite	ZIF-67-Cx/PVDF	PM_0.3_ (NaCl aerosol)	99.461%	≤0.3 μm	32 L/min;	140.235 Pa	>0.0372 Pa^−1^	1476.5 mg g^−1^ for SO_2_ Gas-adsorption capacity (mg g^−1^)	50 ± 5% (30 °C)
[80]	Blend	Silk nanofibers with Ag-doped TiO_2_	PM_2.5_	99.04 ± 1.70%	<2.5 μm	NR	34.3 Pa	0.14 Pa^−1^	98.3% for HCHO (Gas-removal efficiency)	80% (Room temperature)

Notes: NR, not reported in the cited study. Gas-removal efficiency (%) and gas-adsorption capacity (mg g^−1^) are distinct performance metrics and should not be directly compared. Ref. [80] used incense-generated particles in a sealed testing chamber for 20 min at room temperature and 80% relative humidity.

## Data Availability

No new data were created or analyzed in this study. Data sharing is not applicable to this article.

## Abbreviations

The following abbreviations are used in this manuscript:

NF	Nanofiber
NW	Nonwoven
MOF	Metal–organic framework
PAN	Polyacrylonitrile
PVDF	Poly(vinylidene fluoride)
PVA	Poly(vinyl alcohol)
PLA	Polylactic acid
GO	Graphene oxide
rGO	Reduced graphene oxide
ZIF	Zeolitic imidazolate framework
VOCs	Volatile organic compound(s)
AIE	Aggregation-induced emission
TPE-TCF	(E)-2-(3-cyano-5,5-dimethyl- 4-(4-(1,2,2-triphenylvinyl)-styryl)furan-2(5H)-ylidene)malononitrile
SDS	Sodium dodecyl sulfate
TZ-PPP	TPE-TCF/SDS@ZIF-8+ Polyacrylonitrile/Polyvinylpyrrolidone
LB	Lysogeny broth
